# Synchronous Seminoma of Testis and Renal Cell Carcinoma: A Rare Case Report

**DOI:** 10.3390/medicina60091553

**Published:** 2024-09-23

**Authors:** Stasys Auskalnis, Rasa Janciauskiene, Urte Rimsaite, Aurelija Alksnyte, Rasa Ugenskiene

**Affiliations:** 1 Department of Urology, Medical Academy, Lithuanian University of Health Sciences, 44307 Kaunas, Lithuania; 2 Department of Oncology and Hematology, Medical Academy, Lithuanian University of Health Sciences, 44307 Kaunas, Lithuania; 3 Department of Genetics and Molecular Medicine, Medical Academy, Lithuanian University of Health Sciences, 44307 Kaunas, Lithuania

**Keywords:** synchronous, seminoma, renal cell carcinoma, genetic predisposition

## Abstract

*Background and Objectives*: Seminoma is the most common solid malignant tumour in young men. Clear-cell kidney carcinoma is the most common malignancy of the genitourinary tract. However, the synchronous occurrence of both of these tumours is rare. *Case presentation:* We present the case of a 36-year-old patient who presented to a medical facility at the end of 2019 with an enlarged right testicle. A unilateral orchofuniculectomy was performed, and a mass measuring 30 cm was removed. During histological examination, testicular seminoma pT2, R0, was diagnosed. An abdominal computed tomography (CT) scan showed a 6.4 cm × 6.8 cm × 6.7 cm tumour in the right kidney and a metastatic-like lesion in the right adrenal gland. A right nephrectomy and an adrenalectomy and paraaortic and paracaval lymphadenectomies were performed. A histological evaluation confirmed the presence of clear-cell renal carcinoma pT2aR0 G2, adrenal hyperplasia, and seminoma metastases in the removed lymph node. Chemotherapy with a Bleomycin, Etoposide, and Cisplatin (BEP) regimen was carried out. Three years after the last cycle of chemotherapy, a follow-up CT scan showed metastases in the left kidney, the right ischium, and the right lung. A well-differentiated clear-cell carcinoma G1 of the left kidney and metastasis of clear-cell carcinoma G2 in the right ischium were confirmed after the biopsy, and no tumour lesions were found in the lung tissue specimen. Treatment with targeted therapy with Sunitinib was started because the risk was favourable according to the Heng criteria. Genetic testing was performed, and the following genes were analysed: *VHL*, *BAP1*, *CHEK2*, *FH*, *MET*, *MUTYH*, *APC*, and *STK11.* The testing did not reveal any pathogenic or potentially pathogenic mutations or sequence changes of unknown clinical significance in the genes analysed. *Conclusions*: According to the authors, the occurrence of synchronous primary tumours is linked to one’s genetic predisposition. DNA sequencing of tumour tissue could provide more information on the corresponding aetiopathogenesis.

## 1. Introduction

Testicular seminoma is the most common solid malignant tumour in young men and most often clinically manifests as a painless, palpable solid mass. Seminoma predominantly affects younger males aged between 15 and 40 years. Its high prevalence and morbidity at a young age make this tumour a sensitive problem, but it is important to emphasise that seminoma of the testis is often curable even in advanced stages of the disease [1,2]. Previous studies suggest that testicular seminoma may be linked with anomalies in chromosome 12 and disruptions in the spermatogonial niche, leading to germ cell death, infertility, and ultimately testicular seminoma. Somatic mutations in key genes, namely, KIT, GTPase KRAS, and CDC27, may promote this tumour’s development, while mutations in XRCC2 are associated with treatment-resistant cases. These mutations likely contribute to tumour progression and genetic variability [3].

Clear-cell renal carcinoma is the most common type of renal cell carcinoma detected. This tumour is more common in men than in women. Most patients are asymptomatic at the onset of this disease and only become symptomatic when the disease is far advanced [4]. The mortality rate for clear-cell renal carcinoma is 30–40% [5]. Although this tumour is the most common malignancy of the genitourinary tract, the majority of cases are diagnosed incidentally. Early diagnosis of this disease is a critical aspect of effective treatment and efforts to reduce mortality [4]. The best-known predisposing factor for clear-cell renal carcinoma is von Hippel–Lindau (VHL) disease. Familial cases of clear-cell renal carcinoma are mostly associated with VHL germline pathogenic or potentially pathogenic variants. In addition to *VHL*, pathogenic or potentially pathogenic variants in the *MET*, *FLCN*, *TSC1*, *TSC2*, *FH*, and *SDH* genes also contribute to the development of clear-cell renal carcinoma [6].

The combined occurrence of two different neoplastic tumours at the same time is rare. Only a few cases documenting primary testicular cancer occurring synchronously with clear-cell renal carcinoma have been described in the literature [7,8,9]. Synchronous is defined as the occurrence of two or more malignancies that are observed at the same time or within 6 months of each other [10]. The authors outline three main factors that lead to the development of multiple primary cancers: oncogenic mutations, variants in pleiotropic loci spanning different cancer types, and treatment-related induced mutations [11].

We present a clinical case of a testicular seminoma and a synchronous incidentally found clear-cell renal carcinoma.

## 2. Case Presentation

A 36-year-old man arrived at a medical facility at the end of 2019, during the early corona virus (COVID-19) pandemic, with an enlarged right testicle. Upon physical examination, a mass in the testicles measuring approximately 30 cm was observed. Blood tests showed elevated levels of lactate dehydrogenase (LDH) (2191 U/L) and beta-human chorionic gonadotropin (βHCG) (12.95 IU/L), while alpha-fetoprotein (AFP) (3.06 kU/L) levels and renal function were within the normal limits. The patient underwent a unilateral orchofuniculectomy. During the operation, a mass of about 30 cm was radically removed. The conclusion of the histological examination was as follows: testicular seminoma pT2, R0.

**Figure 1:**
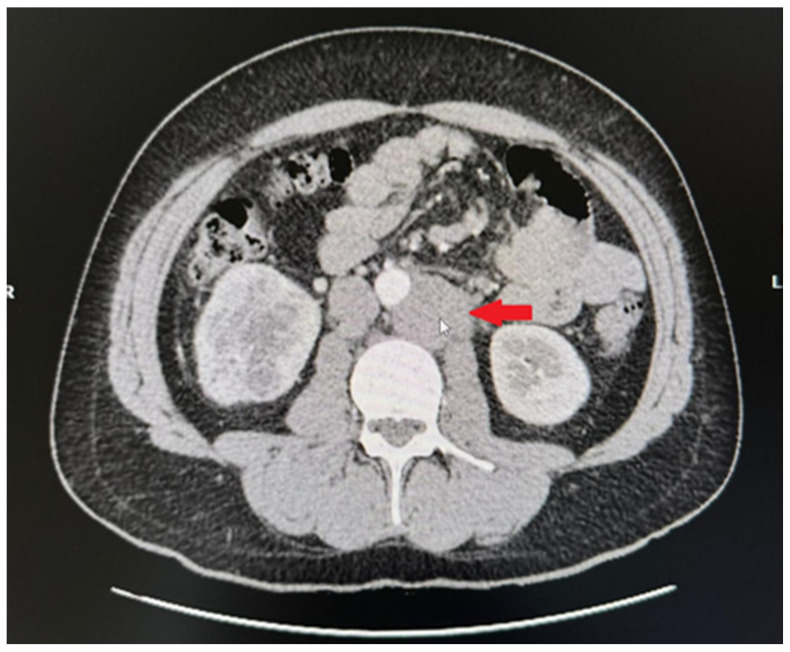
CT image. Pathological paraaortic and paracaval lymph nodes.

**Figure 2:**
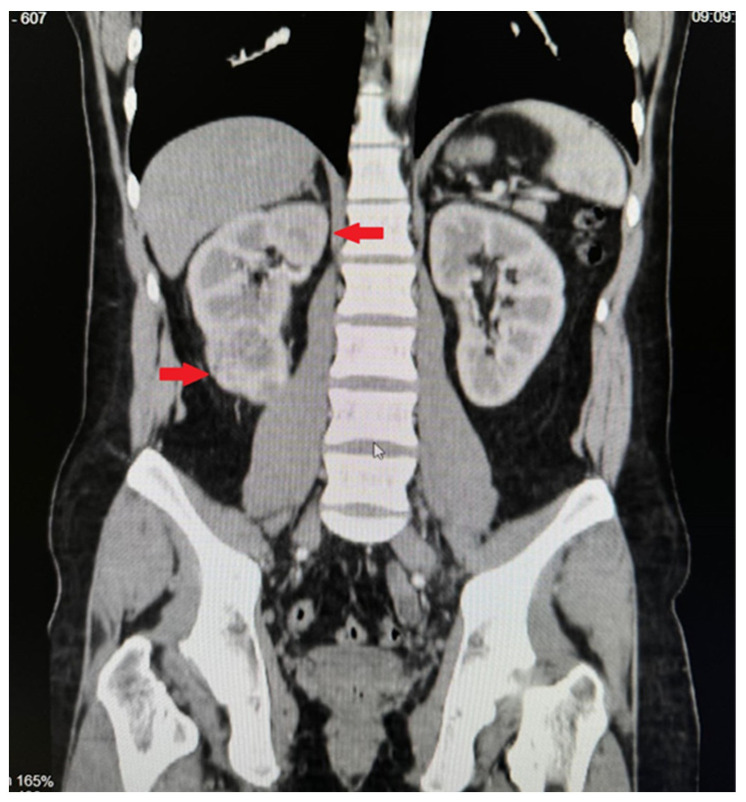
CT image. Tumour mass in the inferior part of the right kidney.

**Figure 3:**
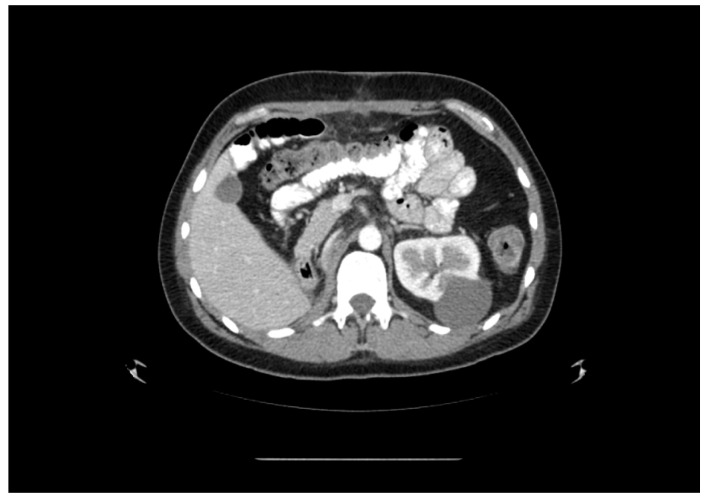
CT image. Post-right-nephrectomy view. Cyst in the left kidney is shown.

Computed tomography of the organs of the chest, abdomen, and pelvis showed pathological paraaortic and paracaval lymph nodes (Figure 1), a tumour measuring 6.4 cm × 6.8 cm × 6.7 cm in size in the inferior part of the right kidney, and a metastatic-like 12 mm × 14 mm lesion in the right adrenal gland (Figure 2). We decided to perform a kidney biopsy. Histological examination confirmed the existence of a well-differentiated clear-cell carcinoma (G1). Surgical treatment was recommended to the patient. During surgery, a right nephrectomy and an adrenalectomy and paraaortic and paracaval lymphadenectomies were performed (Figure 3). Histological examination revealed clear-cell renal carcinoma pT2aR0 G2, seminoma metastases in the removed lymph nodes, and adrenal hyperplasia. Repeated blood tests revealed normal levels of βHCG and AFP, while LDH levels were increased by more than 1.5-fold. The post-operative diagnosis was pure seminoma stage IIIB pT2pN2M0 S2.

The patient was started on a BEP chemotherapy regimen, with four cycles of chemotherapy planned. The patient tolerated the first two cycles of chemotherapy rather well, and no complications were observed. After these two cycles of chemotherapy, a follow-up CT scan of the organs of the chest, abdomen, and pelvis was performed. A CT scan showed no signs of cancer. Treatment was continued, and two cycles of chemotherapy were implemented. After the completion of chemotherapy, further CT scans and follow-ups of tumour markers were recommended.

**Figure 4:**
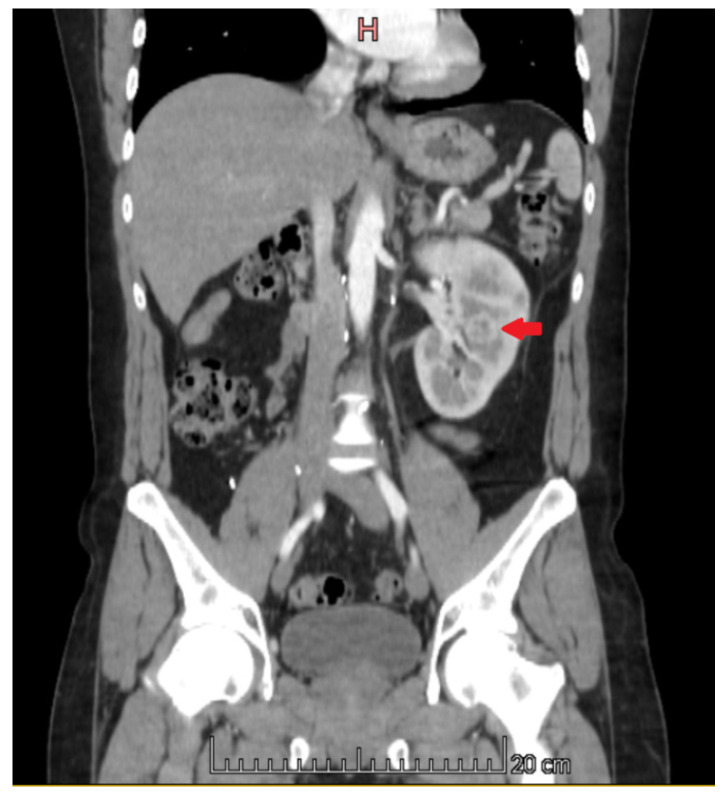
CT image. Metastatic lesions in the left kidney.

**Figure 5:**
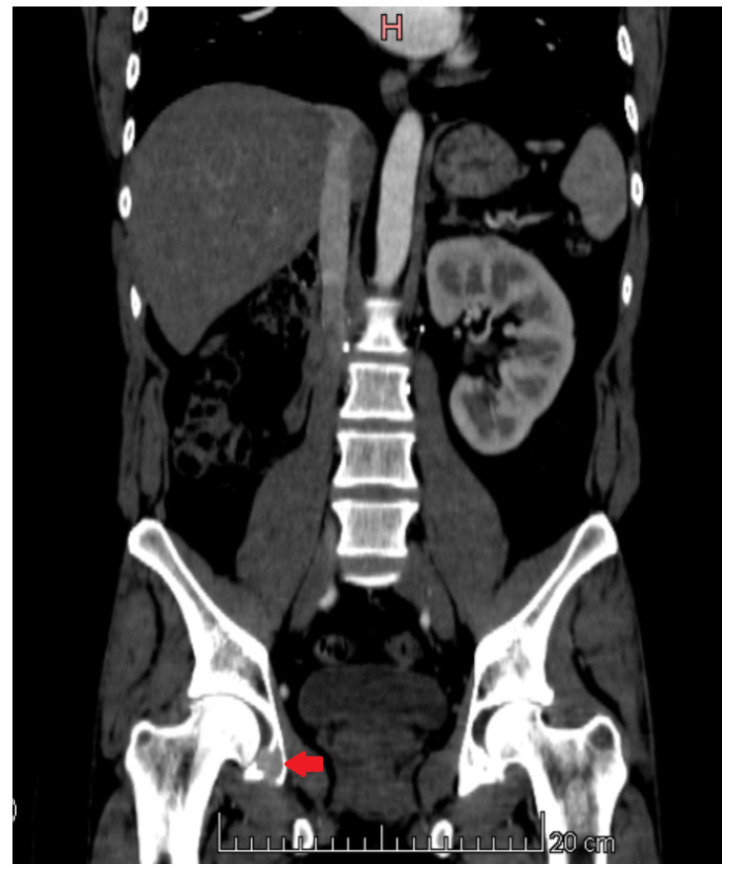
CT image. Osteoclastic-type metastatic tumour in the pelvic bones.

**Figure 6:**
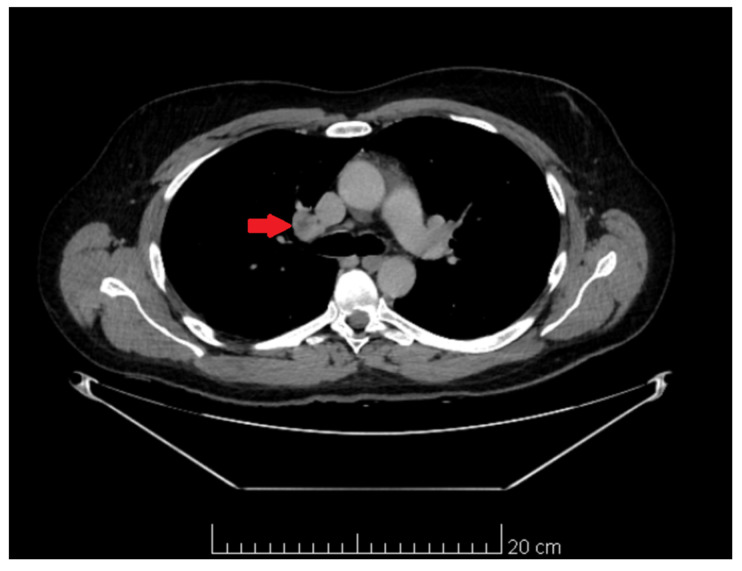
CT image. Pathological lymph node with central necrosis in the hilum of the right lung.

Just over 3 years after the last cycle of chemotherapy, a follow-up CT scan showed progression of the disease. A CT scan of the chest, abdomen, and pelvis showed multiple metastatic tumors measuring up to 2.1 cm × 1.6 cm in the left kidney (Figure 4), osteoclastic metastatic lesions in the pelvic bones (Figure 5), a metastatic lesion approximately 0.6 cm in size in the right lung, and a pathological lymph node measuring 1.6 cm × 1.4 cm with central necrosis in the hilum of the right lung (Figure 6). Tumour markers (LDH, βHCG, AFP, CEA, CA19-9) were within normal limits, and a complete blood count test did not reveal any significant changes.

Upon conducting an ultrasound examination, a slightly hypoechoic tumour of about 2.1 cm × 1.3 cm in size was observed in the dorsal part of the lower half of the left kidney. A biopsy was performed. A histological examination revealed a clear-cell carcinoma of the G1 type. We decided to perform biopsies of the tumours in the right lung and the pelvic bone. A histological examination showed the metastasis of moderately differentiated clear-cell carcinoma (G2) in the pelvic bone, whereas no tumour lesions were found in the lung tissue fragment.

The patient was referred to a medical oncologist for further evaluation and treatment. According to the Heng criteria. the risk group was favourable. The patient’s condition was discussed by a multidisciplinary team, and systemic therapy with Sunitinib 50 mg/day was started.

The patient was given a referral for a consultation with a medical geneticist to assess the aetiology of the synchronous primary tumours. The patient underwent genetic testing. The following genes were analysed: *VHL*, *BAP1*, *CHEK2*, *FH*, *MET*, *MUTYH*, *APC*, and *STK11*. The testing did not reveal any pathogenic or potentially pathogenic mutations or sequence changes of unknown clinical significance in the genes analysed.

To date, the patient has received seven courses of systemic therapy with Sunitinib. According to the CT scan images, the patient shows no signs of disease progression.

## 3. Discussion

About one-third of clear-cell renal carcinoma cases are metastatic at diagnosis. This tumour usually metastasizes to the lungs, bones, liver, and brain. Cases of tumours spreading to the testis have been reported in the literature. Most of them (69.2%) metastasize to the testis ipsilateral to the primary renal cell carcinoma. Metastasis to the testis may resemble a distinct neoplastic process [12].

Testicular seminoma usually metastasizes via the lymphatic route to retroperitoneal lymph nodes and rarely spreads to other areas [13]. Testicular tumours rarely spread to the kidneys, although autopsy studies have shown that up to 25% of non-seminomatous tumours metastasized to the kidney [8]. In a study of 650 patients with testicular seminoma, 2 men had metastases to the kidneys. This study demonstrates that renal metastases of seminoma are rare [13].

In our clinical case, the patient was diagnosed with synchronous tumours of different histological types during the early stages of the COVID-19 pandemic.

A retrospective study conducted in Germany followed patients treated for germ cell cancer of the testis and found that 3.9% of these patients developed a second primary neoplastic process. Interestingly, only 13% of those cases involved synchronous detection of both primary tumours [14]. Similarly, a study conducted in the in the United Kingdom looked at 5555 cases of testicular seminoma and the risk of developing secondary tumours after treatment. After completing treatment for seminoma, three patients were diagnosed with renal cell cancer after 9 years, two patients were so diagnosed after 10–19 years, and two cases were diagnosed with this disease after more than 20 years [15].

Treatment of testicular cancer is associated with a small but clearly identifiable risk of developing secondary solid tumours. The incidence of secondary neoplasia is relatively low and therefore does not influence the choice of treatment strategy [16].

Metachronous malignancies occurring more than 6 months after a diagnosis of testicular cancer are uncommon, and the development of renal cell carcinoma after a previous testicular tumour is extremely rare [14]. The literature describes only 12 clinical cases of patients with testicular and renal malignancies. Among these, only one case report describes a synchronous presentation of testicular cancer (a mixed germ cell tumour) and clear-cell kidney carcinoma [7,8,9].

According to the authors, the multiple neoplastic process is linked to the influence of genetic factors [8,14]. The *VHL* gene is a tumour suppressor gene that plays an important role in regulating the hypoxia pathway in the sporadic pathogenesis of clear-cell renal carcinoma. Alterations in the *VHL* gene are detected in 50–70% of clear-cell renal carcinoma cases, but they are not associated with overall survival [17]. The *BAP1* (BRCA1-associated protein 1) gene is a tumour suppressor gene that encodes a deubiquitinating enzyme. The latter is responsible for the cell cycle, differentiation, transcription, and the response to DNA damage. Clinical studies have shown that the *BAP1* gene is lost or inactivated in various cancers. Recent studies have shown that variants in the *BAP1* gene cause a tumour susceptibility syndrome, which is characterised by an increased incidence of early tumours. It increases the risk of mesothelioma, clear-cell renal carcinoma, and other malignancies [18,19]. The *CHEK2* (Checkpoint Kinase 2) gene is important for maintaining genome integrity and regulating the cell cycle. In one study, 7.5% of patients with clear-cell renal carcinoma had variants in this gene, but did not exhibit any distinct characteristics. Patients with pathogenic and potentially pathogenic variants of the *CHEK2* gene were observed to develop a second neoplastic process many years later [6]. The *FH* gene change leads to a rare aggressive subtype of clear-cell renal carcinoma caused by an inherited or sporadic loss-of-function variant of the *FH* gene. It has been shown that 15% of people with the *FH* gene variant will develop clear-cell renal carcinoma [20]. The cited authors report that *VHL*, *BAP1*, *FH*, and *MET* are specific genes that increase the risk of developing renal cell cancer, whereas *CHEK2*, *MUTYH*, *APC*, and *STK1* are not typically associated with the development of renal tumours [21]. Variants in the *BRCA1/2* and *CHEK2* genes impair the response to and repair of DNA damage, resulting in a compensatory increase in the activity of the DNA single-strand annealing (SSA) repair system. The latter, due to its propensity to generate DNA repair errors, leads to genomic instability and increases the risk of second malignancy development [11].

In addition to genetic predisposition, the occurrence of multiple primary tumours in patients with testicular germ cell cancer is also thought to be due to radiation exposure or chemotherapy treatment [8,14].

## 4. Conclusions

This clinical case of synchronous primary testicular and renal cancers is a rare occurrence. Such a unique case may not only pose a diagnostic dilemma but also raise questions about the choice of appropriate treatment. DNA sequencing of tumour tissue could provide more information on the aetiopathogenesis of such synchronous primary tumours. Both malignant tumours should be treated according to the established guidelines, depending on the clinical stage of the disease.

## Data Availability

The raw data supporting the conclusions of this article will be made available by the authors, without undue reservation.
