# Correction: Auskalnis et al. Synchronous Seminoma of Testis and Renal Cell Carcinoma: A Rare Case Report. *Medicina* 2024, *60*, 1553

**DOI:** 10.3390/medicina60122042

**Published:** 2024-12-11

**Authors:** Stasys Auskalnis, Rasa Janciauskiene, Urte Rimsaite, Aurelija Alksnyte, Rasa Ugenskiene

**Affiliations:** 1 Department of Urology, Medical Academy, Lithuanian University of Health Sciences, 44307 Kaunas, Lithuania; 2 Department of Urology, Hospital of Lithuanian University of Health Sciences Kauno Klinikos, 44307 Kaunas, Lithuania; 3 Department of Oncology and Hematology, Medical Academy, Lithuanian University of Health Sciences, 44307 Kaunas, Lithuania; 4 Department of Genetics and Molecular Medicine, Medical Academy, Lithuanian University of Health Sciences, 44307 Kaunas, Lithuania

## Additional Affiliation

In the published publication [1], there was an error regarding the affiliation for “Stasys Auskalnis and Aurelija Alksnyte”. In addition to affiliation 1, the updated affiliations should include: “Department of Urology, Hospital of Lithuanian University of Health Sciences Kauno Klinikos, 44307 Kaunas, Lithuania”. The authors state that the scientific conclusions are unaffected. This correction was approved by the Academic Editor. The original publication has also been updated.
